# Assessment the effect of vitamin D supplementation on plasma vitamin D levels, inflammation, and oxidative stress biomarkers based on vitamin D receptor genetic variation in breast cancer survivors: a protocol for clinical trial

**DOI:** 10.1186/s41043-021-00272-9

**Published:** 2021-11-02

**Authors:** Elham kazemian, Mohammad Esmaeil Akbari, Nariman Moradi, Safoora Gharibzadeh, Atieh Amouzegar, Laura S. Rozek, Alison M. Mondul, Maryam Khademolmele, Katie R. Zarins, Nasim Ghodoosi, Zahra Shateri, Soudabeh Fallah, Sayed Hossein Davoodi

**Affiliations:** 1 Non-Communicable Diseases Research Center, Alborz University of Medical Sciences, Karaj, Iran; 2 Cancer Research Center, Shahid Beheshti University of Medical Sciences, Tehran, Iran; 3 Department of Biochemistry, Faculty of Medicine, Kurdistan University of Medical Sciences, Sanandaj, Iran; 4 Department of Epidemiology and Biostatistics, Pasteur Institute of Iran, Tehran, Iran; 5 Endocrine Research Center, Research Institute for Endocrine Sciences, Shahid Beheshti University of Medical Sciences, Tehran, Iran; 6 Department of Environmental Health Sciences, University of Michigan, Ann Arbor, MI USA; 7 Department of Epidemiology, University of Michigan School of Public Health, Ann Arbor, MI USA; 8 Department of Nutrition Science, Faculty of Medical Science and Technology, Science and Research Branch (SRBIAU), Islamic Azad University, Tehran, Iran; 9 Department of Community Nutrition, School of Nutritional Sciences and Dietetic, Tehran University of Medical Sciences, Tehran, Iran; 10 Department of Biochemistry, Faculty of Medicine, Iran University of Medical Sciences, Tehran, Iran; 11 National Nutrition and Food Technology Research Institute, Faculty of Nutrition Sciences and Food Technology, Shahid Beheshti University of Medical Sciences, Shahrake Gharb, No. 7, Hafezi St. Farahzadi Blv, Tehran, Iran

**Keywords:** Breast cancer survivors, Vitamin D receptor, Vitamin D, Simultaneously

## Abstract

**Background:**

Both human genes and environmental exposures, due to complex interplay, play important role in the cancer etiology. Vitamin D is associated with a reduced risk of incidence and mortality of several human cancers. This study will aim to investigate the possible effects of individual polymorphisms in vitamin D receptor (*VDR*) as well as effects of *VDR* haplotypes on response to vitamin D supplementation in breast cancer survivors.

**Methods:**

This is an interventional study in which the effects of vitamin D supplementation on plasma vitamin D levels, inflammatory and antioxidant biomarkers and factors associated with cell proliferation, differentiation, damage, and apoptosis will be investigated stratified by variations in *VDR* genotype. The present study will be conducted on breast cancer survivors referred to the Shohadaye Tajrish hospital and its associated clinics. One hundred ninety-eight breast cancer survivors will receive 4000 IU of vitamin D3 daily for 12 weeks. *VDR* Fok1, ApaI, TaqI, BsmI, and Cdx-2 genotype will be determined at the end of the study and responses to vitamin D supplements (inflammatory, antioxidant, cell proliferation, differentiation, damage, and apoptosis biomarkers) will be compared between the three subgroups of each *VDR* polymorphism as well as different *VDR* haplotype categories.

**Discussion:**

Genetic variation is a fundamental factor influencing individuals’ divergent responses to diet, nutritional status, metabolic response, and diet-related health disorders. Furthermore, studies of gene and environment interactions will provide a precise and accurate assessments of individuals’ dietary requirements by considering both the genetic and environmental aspects simultaneously. The results of the current study, to some extent, will highlight the discrepancies existing in the findings of different studies regarding vitamin D, *VDR*, and cancer by considering both the genetic and environmental aspects simultaneously. If responses to vitamin D supplementation could be modified by *VDR* SNPs, determining the distribution of *VDR* polymorphisms in both breast cancer survivors and healthy populations will provide a new insight into the vitamin D requirements of individuals to prevent cancer and its related mortality based on their genotypes.

*Trial registration* This trial has been registered on Iranian Registry of Clinical Trials (IRCT) under the identification code: IRCT2017091736244N1, registration date: 2017-11-10, http://www.irct.ir/trial/27153

## Background

Cancer is one of the major multifactorial health problems over the last decades due to increased life span, the epidemiologic transition, and individuals being exposed to various risk factors for a long period [1]. Breast cancer is one of the most frequently diagnosed malignancies, accounting for 25.1% of all tumors, and is the second leading cause of cancer death in women worldwide [1, 2]. Higher incidence rates of breast cancer have also been reported in developed countries [1]. Risk factors for developing breast cancer include increasing age, family history of first-degree relatives with breast cancer, race, the mutation in *BRCA1* and *BRCA2* genes, menarche history, nulliparity, breast characteristics, hormone use, alcohol and tobacco use, dietary habits, physical activity, and obesity [2, 3].

Low vitamin D is associated with an incidence and mortality of several cancers [4, 5]. Increased risk for up to 15 prevalent cancer, including breast carcinoma, has been documented by several epidemiologic studies [4, 6–9]. Although the exact mechanisms by which vitamin D3 reduces the risk of cancer are unknown, it has been postulated that 1,25-dihydroxy vitamin D3 (1,25(OH)2D3), the active form of vitamin D3, has anti-proliferative, anti-angiogenic, pro-apoptotic [10], and anti-inflammatory properties [11] in many cells, including breast cancer cells. In addition, terminal cell proliferation, induced by 1,25(OH)2D3, resulted in declining growth and spread of malignant cells growth and spread [12].

1,25(OH)2D3 is locally produced in mammary tissue via conversion of circulating 25-hydroxyvitamin D3(25(OH)D) to 1,25(OH)2D3 by endogenous 1-α-hydroxylase in the breast [13]. The physiological effects of vitamin D3 are mostly modulated via the binding of 1,25–1,25(OH)2D3 to the vitamin D receptor (*VDR*) [14]. *VDR*, a nuclear hormone receptor superfamily member, encoded by a large gene located on chromosome 12cen-q12, containing at least five promoter regions, eight coding exons, and six untranslated exons [15]. *VDR* has been identified in many organs, including normal breast tissue and most breast tumors [16]. Transcription of several genes involved in cellular growth, differentiation, apoptosis, angiogenesis, inflammation [17], and metastasis is regulated via *VDR* [16].

Anticancer properties of vitamin D_3_ postulated that sequence level variations in the *VDR* gene may alter breast cancer risk either exclusively or via gene–gene or gene–environment interactions [18]. Findings of preliminary studies indicate that allelic differences in Fok1, Bsm1, Taq1, Apa1, EcoRV, and Cdx2 were the most frequent single-nucleotide polymorphisms (SNPs) correlated with malignancies [17, 19]. Meta-analysis studies on the association between *VDR* polymorphisms and breast cancer risk are inconsistent [19–24]. However, data from descriptive studies in which various effects of environmental exposures, e.g., vitamin D intake, plasma vitamin D status, etc., on breast cancer risk in persons with different genotypes were overlooked, which may account for discrepancies observed among the studies.

This will be the first study designed to evaluate the possible role of individual polymorphisms in *VDR* and to conduct an extensive analysis of *VDR* haplotypes on different aspects of responses of breast cancer survivors, i.e., inflammatory, oxidative stress, and metabolic biomarkers, biomarkers associated with cell proliferation, differentiation, damage, apoptosis, and anthropometric measures to daily supplementation of 4000 international unit (IU) vitamin D for 12 weeks.

## Objectives

### Primary objectives

1. To compare plasma 25-hydroxy vitamin D_3_(25(OH) D_3_) within and between polymorphic groups (Fok1, Bsm1, Taq1, Apa1, and Cdx2) in breast cancer survivors, adjusted for potential confounders.
2. To compare plasma 25-hydroxy vitamin 25(OH) D_3_within and between haplotype groups in breast cancer survivors, adjusted for potential confounders.

### Secondary objective

1. To compare inflammatory, oxidative stress, and metabolic biomarkers, biomarkers associated with cell proliferation, differentiation, damage, apoptosis, and anthropometric measures within and between polymorphic groups (Fok1, Bsm1, Taq1, Apa1, and Cdx2) in breast cancer survivors, adjusted for potential confounders**.**
2. To compare inflammatory, oxidative stress, and metabolic biomarkers, biomarkers associated with cell proliferation, differentiation, damage, apoptosis, and anthropometric measures within and between haplotype groups in breast cancer survivors adjusted for potential confounders.
3. To perform an investigational analysis of mean changes in inflammatory, oxidative stress, and metabolic biomarkers, biomarkers associated with cell proliferation, differentiation, damage, apoptosis, and anthropometric measures based on VDR single-nucleotide polymorphisms (SNPs), i.e., Fok1, Bsm1, Taq1, Apa1, and Cdx2.

To perform an investigational analysis of mean changes in inflammatory, oxidative stress, and metabolic biomarkers, biomarkers associated with cell proliferation, differentiation, damage, apoptosis, and anthropometric measures in different haplotype groups.

### Hypothesis

1. Daily supplementation of vitamin D3 will ameliorate circulating 25(OH)D concentrations in breast cancer survivors.
2. Daily supplementation of vitamin D3 will improve inflammatory, oxidative stress, and metabolic biomarkers, biomarkers associated with cell proliferation, differentiation, damage, apoptosis, and anthropometric measures in breast cancer survivors.
3. Response to daily supplementation of vitamin D3 will vary between different *VDR* polymorphic groups.
4. Response to daily supplementation of vitamin D3 will vary between different *VDR* haplotype groups.

## Methods/Design

### Study design

This will be a 3-month clinical trial conducted on breast cancer survivors to assess the potential effects of vitamin D3 supplement on plasma vitamin D levels, inflammatory and antioxidant biomarkers, and factors associated with cell proliferation, differentiation, damage, and apoptosis based on polymorphic variation in VDR gene. The intervention period will be limited to the winter months (February, April) and spring months (May, June) to reduce dermal vitamin D synthesis. The current study will be accomplished by collaboration between the Faculty of Nutrition Sciences and Food Technology and the National Nutrition and Food Technology Research, the Cancer Research Center, and the Research Institute for Endocrine Sciences, Shahid Beheshti University of Medical Sciences.

### Participants

This study will be conducted on women previously diagnosed with invasive or in situ breast cancer, whose cancers were confirmed by pathological examination. Patients admitted to Shohadaye Tajrish hospital and its associated clinics in Tehran who had been diagnosed with cancer for at least the past six months and their treatments, including surgery, radio, and chemotherapy had been completed will be requested to participate in the study.

#### Inclusion criteria

1. Patients with breast cancer including invasive or in situ carcinoma whose cancer had been confirmed by pathological examination
2. Patients diagnosed with breast cancer at least 6 months prior to the study and treatment protocol including surgery, radio, and chemotherapy was completed
3. Women aged 25–65 years
4. BMI between 18.5 and 35 kg/m^2^
5. No use of vitamin D_3_ supplements, i.e., 1000 IU daily or 50,000 IU weekly or 300,000 intramuscular injections, for at least 4 months prior to the study.
6. No use of dietary, herbal, or omega-3 supplements during the intervention period

#### Exclusion criteria

1. History of malabsorption syndrome, calcium metabolism disorders, gastrointestinal, renal, inflammatory (sarcoidosis, etc.), and other endocrinological diseases
2. Under treatment for weight reduction
3. High levels of plasma vitamin D
4. Pregnancy

### Sample size calculation

**Fig. 1:**
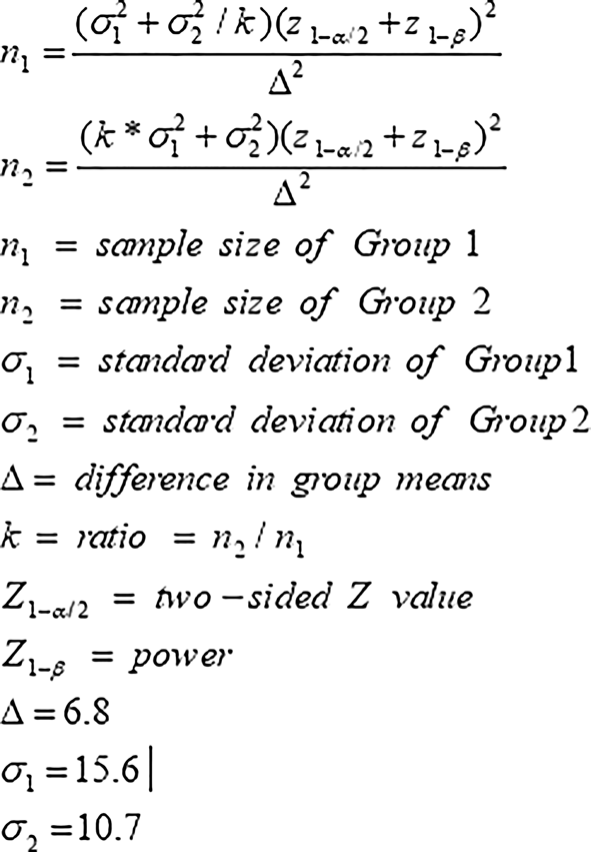
Sample estimation formula and assumption

Sample size will be calculated by Epi Info software, using the following formula and assumption (Fig. 1). We will have 55 subjects per treatment group. Considering the third polymorphic category and the 20% dropout rate during 12 weeks, 198 subjects will be recruited.

It should be mentioned that the global minor allele frequency (MAF) for Fok1 is 0.3285; the calculated sample size will result in an allocation of appropriate samples in each of the polymorphic groups.

#### Intervention

One hundred ninety-eight breast cancer survivors will receive 4000 IU of vitamin D_3_ daily for 12 weeks (cholecalciferol tablets will be manufactured by *JALINOUS PHARMACEUTICAL* CO, Tehran, Iran). Subjects will be given 84 pills at the beginning of the study and will be instructed to consume one tablet with one of their daily meals. Participant compliance will be assessed through biweekly telephone calls. The intervention period will be 12 weeks. Health-related benefits of vitamin D without any increase in the risk of health problems have been demonstrated by plasma 25(OH)D levels of 75–110 nmol/l, achieved through daily supplementation of vitamin D_3_ in the range of 1800–4000 IU [25]. Anthropometric measurement, body composition analysis, dietary, physical activity, and laboratory assessments will be carried out before and after supplementation, and outcomes of interest will be compared between variant *VDR* polymorphic groups.

#### Compliance

All subjects will be given a leaflet with instructions on how to use vitamin D tablets. Participants’ adherence to the supplementation regimen will be assessed biweekly by self-report through telephone calls. Subjects will be requested to bring their empty box of pills back at the end of the study. Adherence will be calculated by the number of pills consumed divided by the numbers prescribed during the study and presented as a percentage of adherence to supplementation. Subjects will be excluded if they had not consumed over 10% of tablets.

### Study measurements

#### Investigation of general and demographic information

Demographic data including age, education, marital status, smoking, history of drug and alcohol use, oral contraceptive pill (OCP) usage, treatment protocol used for individual breast cancer, i.e., surgery, chemo, radio, and hormone therapy, will be obtained both through interview and review of medical records.

#### Pathologic assessment

Background and pathologic data including estrogen receptor, progesterone receptor, and human epidermal growth factor receptor 2(HER2) expression, histologic grade, tumor size, tumor stage and grade, lymph node involvement, metastasis and surgery, radio, chemo, and hormone therapy information will be obtained from medical records.

#### Anthropometrics measurements

Height will be measured using the Seca stadiometer to the nearest 0.1 cm, with subjects facing directly ahead without shoes, feet together, and arms by the sides. Body weight will be measured with light clothes, without shoes, using a digital scale to the nearest of 0.1 kg. BMI will be calculated by dividing weight (kg) by height (m) squared. Waist circumference (WC) will be measured over light clothing at the mid-point between the highest point of the iliac crest and the last floating rib using a measuring tape to the nearest of 0.1 cm. Hip circumference (HC) will also be measured by an unstretched tape measure to the nearest of 0.1 cm; will be placed around the point with the maximum circumference over the buttocks. For both measurements, participants will stand with feet fairly close together with weight equally distributed on each leg and minimal clothing; measurements will be taken after a gentle expiration.

#### Body composition assessment

Body fat percentage and distribution will be assessed by bioelectrical impedance analysis (BIA) (Tanita BC-418, Illinois, USA).

#### Physical activity assessment

Physical activity will be measured by the International Physical Activity Questionnaire (IPAQ) comprising seven questions translated by Iran's National Elites Foundation; overall physical activity will be reported as Metabolic Equivalent Task minutes per week (MET-min/wk), calculated as follows: [26]

- Walking MET-minutes/week = 3.3 * walking/minutes per day * number of days per week spent on walking was reported.
- Moderate MET-minutes/week = 4.0 * moderate intensity/minutes per day * number of days per week spent on moderate intensity activity
- Vigorous MET- minutes per week = 8.0 * Vigorous intensity/minutes per day * number of days per week spent on vigorous intensity activity
- Total physical activity MET-minutes/week = Walking + Moderate + Vigorous MET minutes/week scores

#### Sun exposure assessment

Sun exposure will be determined by a questionnaire designed for this purpose. Participants will be asked to report hours per week spent outdoor activity, and body surface area (BSA) exposed to sunlight while outdoors. The total percentage of BSA exposed to sunlight will be calculated as the sum of the reported percentage of BSA exposed reported for each part of the body by subjects. BSA will be regarded as follows: 9% for face, 1% for each hand, 9% for each arm, and 18% for each leg. Some parts of the body including chest waist and abdomen will not be considered in the total BSA calculation.

A sun exposure index will be finally calculated by multiplying the percentage of BSA exposed by the hours of exposure to sunlight per week [27].

#### Dietary intake assessment

A twenty-four-hour recall for three days, including a weekend day and two working days, will be used to assess dietary intakes at the beginning and end of the study. Participants will be asked to record the total consumption of foods, beverages, and supplements during 24 h based on standard household measures, including tablespoons and cups; they will be given a leaflet describing detailed instructions on recording their intakes.

### Laboratory assessment

#### Blood sampling

Fasting venous blood samples (15 ml) will be taken in spring (April–May) and winter (February–March) from all participants in a sitting position by phlebotomy. Blood sample will be divided in two tubes: (1) a plastic tube with an anticoagulant, EDTA (Ethylenediaminetetraacetic acid); (2) a plastic tube with an anticoagulant, EDTA and anti-protease. Blood collection tubes will be tagged with identification codes; the tubes containing the EDTA blood sample will be centrifuged at 3000×*g* for 10 min for white blood cell (WBC) separation, while the tubes containing EDTA and anti-protease blood sample will be centrifuged for plasma separation. Obtained plasma will be distributed in clean micro-tubes and will be kept at − 80 °C until the day of analysis. White blood cells will be transferred to clean micro-tubes in aliquots, and they will also be kept at − 80 until the day of examination.

#### DNA extraction

DNA will be extracted from WBC using the DNA Blood Kit. To determine DNA concentration, spectrophotometric absorbance of the samples at 260 nm and 280 nm will be measured using a Nanodrop spectrophotometer thermo scientific. DNA quality will then be determined by the A260/A280 ratio, and an A260/A280 ratio of 1.7–2 will be considered as good quality DNA. Extracted DNA will subsequently be stored at − 20 °C for the following PCR amplification of and restriction fragment length polymorphism (RFLP) and Tetra-amplification refractory mutation system (ARMS) PCR.

#### DNA genotyping

Polymerase chain reaction (PCR)-RFLP methods will be used for *VDR* genotyping at FokI, ApaI, TaqI, BsmI SNPs, and tetra-primer ARMS-PCR method for Cdx-2. Initial PCR amplification will be performed in a total volume of 50 μl containing 100–300 ng of genomic DNA, 1 pmol of each forward and reverse primer, 25 μl of master mix PCR. DNA samples will be amplified using T100 thermal cycler with cycling parameters as follows: initial denaturation at 95 °C for 5 min, 30 cycles at 94 °C for 45 s for denaturation, 45 s at 60 °C for annealing, and 60 s at 72 °C for extension, will be followed by 7 min at 72 °C for final extension and cooling at 12 °C for 10 min. Two percent agarose gel electrophoresis will be conducted to determine the accuracy of amplified fragment of DNA.

FokI, ApaI, TaqI, BsmI polymorphisms of the *VDR* gene will be detected by RFLP as follows:

Fok1: RFLP will be performed in 40-μl mixture containing 1 μl of restriction endonuclease FokI, 2 μl 10X buffer FokI, 17 μl nuclease-free water, and 10 μl amplified PCR product. The incubation temperature will be adjusted based on the manufacturer's instructions for the restriction endonuclease Fok1.

ApaI: RFLP will be carried out in a tube containing 2 μl of restriction endonuclease ApaI, 2 μl 10X buffer ApaI, 18 μl nuclease-free water, and 10 μl amplified PCR product. The incubation temperature will be adjusted based on the manufacturer's instructions for the restriction endonuclease ApaI.

TaqI: RFLP will be performed in a micro-tube containing 1 μl of restriction endonuclease TaqI, 2 μl 1X buffer TaqI, 18 μl nuclease-free water, and 10 μl amplified PCR product. The incubation temperature will be maintained based on the manufacturer's instructions for the restriction endonuclease. Micro-tubes will completely be sealed with parafilm to avoid evaporation.

BsmI: RFLP will be performed in 40-μl mixture containing 1 μl of restriction endonuclease BsmI, 2 μl 1X buffer BsmI, 18 μl nuclease-free water, and 10 μl amplified PCR product. The incubation temperature will be maintained based on the manufacturer's instructions for the restriction endonuclease.

Digested products will be resolved on a 3% agarose gel containing 0.5 μg/mlTBE 0.5X using a gel electrophoresis system at 100 V for 90 min. The gel will be visualized under UV light using Gel Documenting system.

Cdx2 polymorphism of *VDR* will be determined using tetra-primer ARMS-PCR. The genomic DNA will be amplified by four primers in a single PCR carried out in a total volume of 50 μl, containing 100–300 ng of genomic DNA, 25 μl of master mix PCR, and 1 pmol/μl of each primer followed by electrophoresis. The 3′ end of two primer pairs will be specified to G allele and the other for A allele.

Sequencing of 10% of samples will be carried out to validate the accuracy of genotype scoring by RFLP-PCR and tetra-primer ARMS-PCR.

#### Biochemical assay

Lipid profiles including plasma total cholesterol (TC), low-density lipoprotein cholesterol (LDL-C), high-density lipoprotein cholesterol (HDL-C), triglycerides (TG), alanine aminotransferase (ALT), and aspartate aminotransferase (AST) will be measured using the enzymatic calorimetric method. Plasma 25(OH)D concentrations will be measured by ELISA kit.

#### Inflammatory biomarker measurement

Plasma inflammatory markers will be evaluated by measuring plasma interleukin 6 (IL6), tumor necrosis factorα (TNFα), human high sensitivity C-reactive protein (hs-CRP), and interferon β (IFNβ) levels.

#### Oxidative stress assessment

Oxidative stress will be determined by measurement of plasma superoxide dismutase (SOD), total antioxidant capacity (TAC), and malondialdehyde (MDA), using colorimetric method [28, 29].

#### Cell proliferation, differentiation, damage, and apoptosis assessment

Apoptosis will be assessed by determining plasma Bcl2 levels. The 8-hydroxy-2'-deoxyguanosine (8-OHdG) will be measured as DNA damage indicator using ELISA. Also, ELISA method will be implemented to determine plasminogen activator inhibitor-1(PAI-1), E-cadherin, matrix metallopeptidase 9 (MMP9), soluble intercellular adhesion molecule-1 (sICAM-1), and soluble vascular cell adhesion molecule-1 (sVCAM-1) as a proliferation, differentiation, and metastasis status.

### Statistical analyses

We will check the distribution of variables using the Shapiro–Wilk test. The genetic factors, Apa-I, Fok1, Taq-I, Bsm1, and Cdx2, will be considered as the main exposures. To check the within-factor differences in case of normality, we will use one-way ANOVA, and for skewed (non-normal) variables, we will use the Kruskal–Wallis *H* test.

To assess the effect of each category of exposures on outcomes of interest, we will use multiple linear regression. We will log-transform all of the non-normal variables and compute the differences for all response variables (25(OH)D3, BMI, WC, HC, fat mass, TNFα, IL6, hs-CRP, PAI-1, Bcl2, SOD, TAC, MDA, 8-OHdG, MMP9, IFNβ, E-cadherin, sICAM, and sVCAM,) from second and baseline follow-up. All analyses will be adjusted for potential confounders, including energy, fat, carbohydrate and protein intakes, physical activity, sun exposure, and 25(OH)D3 at baseline. The significant effect of the coefficients of different categories of genetic factors will be checked; we will use cluster robust standard errors because of repeated measurements on each subject.

Deviation from Hardy–Weinberg equilibrium for each SNP will be determined using Fisher’s exact test, performing by the HWE exact function in the R package “genetics.” Deviation from Hardy–Weinberg equilibrium for each SNP will be determined using “genhwi” command in Stata. Haplotypes frequencies and distributions will be estimated using the Haplo.em, the haplo.stats software package in the R [30]. The association of haplotypes with outcomes of interest will be tested using the means of the haplo.glm function of the R package “haplo.stats.”

We will use Stata 14.0 (StataCorp. 2015. Stata Statistical Software: Release 14. College Station, TX: StataCorp LP.), and R Statistical software (R Core Team (2017). R: A language and environment for statistical computing. R Foundation for Statistical Computing, Vienna, Austria. URL https://www.R-project.org/.).

### Approval and ethical considerations

The protocol was approved by the National Nutrition and Food Technology Research Institute (NNFTRI), Shahid Beheshti University of Medical Sciences (SBUM), Tehran, Iran (IR.SBMU.NNFTRI.REC.1395.62) (Registration ID in IRCT: IRCT2017091736244N1, registration date: 10-11-2017, http://www.irct.ir/trial/27153). All participants will be requested to sign a written informed consent at recruitment, in which trial procedures will be described. Participants will be informed that they have the right to withdraw from the study at any time.

## Discussion

The global cancer burden is expected to escalate about 47% by 2040, raising concerns regarding cancer prevention strategies [31]. The complex interplay between human genes and environmental exposures significantly contributes to the etiology of cancer. Dietary pattern and nutrients intake, as well as quantity and quality of diet, are reported to play a role in cancer development [32]. It has been indicated that the risk for developing cancer varies among individuals with similar dietary intakes, which could partly be explained by genetic variation in the form of SNPs, the most common genetic variation involved in nutrient genetic heterogeneity [32, 33]. Although pharmacogenetic testing, using gene polymorphism data to estimate drug transportation, metabolization, and requirements, has been applied to predict the efficiency of drugs, similar procedures have not been employed with nutrigenetics [34]. The study of genes and nutrition interaction will establish personalized nutrition that can enhance public health dietary recommendations [32]. It is believed that comprehension of genetic-based individualized response to dietary components will result in a more precise understanding of the exposure of intended tissue to dietary factors and their metabolites [35]

The strongest evidence for an association between genetic polymorphisms in VDR and health-related outcomes originates from the study by Abrams et al., in which the association between Fok1 polymorphism of *VDR* and bone mineral metabolism was documented [36]. Wong et al. in 2003 reported that individuals with the VDR Fok1 f allele manifested an elevated risk for colorectal cancer when dietary calcium intake is low [37]. It has been demonstrated that the *VDR* Fok1 f allele was associated with decreased calcium accumulation and increased bone problems [38]. Diets are a mixture of protective, carcinogenic, and mutagenic compounds metabolized via different types of enzymes in the human body [33]. Likewise, the effects of dietary elements on cancer risk may be modified by variations in genes involved in their transport and metabolism [39]. Millions of SNPs have been identified to modify multiple mutual relations of diet, genes, and health-related problems [40].

Most previous studies focused only on environmental aspects or the gene alone, but not on both simultaneously [33]. Hence, future studies using nutrigenomic approaches focused on genetic variation, diet, lifestyle, and environment to develop personalized nutritional strategies for cancer prevention and treatment are urgently needed.

To the best of our knowledge, this will be the first clinical trial being conducted to assess the role of *VDR* polymorphisms in individuals’ responses to vitamin D supplementation through assessing different aspects of cancer etiology, e.g., inflammatory, oxidative stress and metabolic biomarkers, biomarkers associated with cell proliferation, differentiation, damage, apoptosis, and anthropometric measures. Investigating different types of biomarkers will enable us to study the potential mechanisms and pathways through which vitamin D will be involved in cancer etiology and its related milieu. Response to vitamin D based on *VDR* polymorphisms will also be allowing us to scrutinize the gene and environment interactions simultaneously. The following will be the strengths of our study: first, the intervention will be carried out in the cold months, i.e., winter and early spring, when the endogenous synthesis of vitamin D is very low and due to the high prevalence of vitamin D deficiency in Tehran inhabitants [41], more noticeable response to vitamin D supplementation will be anticipated. Second, the results of the present study may illustrate the discrepancies that exist in the findings of different studies regarding vitamin D, VDR, and cancer by considering both genetic and environmental aspects simultaneously. If the response to vitamin D supplementation could be modified by VDR SNPs, determining the distribution of VDR polymorphisms in both breast cancer survivors and healthy populations will provide a new, much-needed insight on individual’s vitamin D requirements to prevent cancer and its related mortality based on the genotypes of these individuals. However, our study will be limited by the relatively small number of SNPs checked, which will likely represent only a small fraction of the variation in the VDR. In addition, the small sample size of the current investigation may result in lower statistical power. Furthermore, the 12-week trial may not be long enough to detect the anti-carcinogenic effects of vitamin D3. Finally, an absence of a control group will make it very difficult to attribute long-term changes in tumor markers to vitamin D3 per se.

## Data Availability

The datasets used during the current study will be available from the corresponding author on reasonable request.

## Abbreviations

BMI: Body mass index
VDR: Vitamin D receptor
IRCT: Iranian Registry of Clinical Trials
SNPs: Single-nucleotide polymorphisms
IU: International unit
25(OH) D_3_: 25-Hydroxy vitamin D_3_
MAF: Global minor allele frequency
OCP: Oral contraceptive pill
HER2: Human epidermal growth factor receptor 2
WC: Waist circumference
HC: Hip circumference
BIA: Bioelectrical impedance analysis
IPAQ: International Physical Activity Questionnaire
BSA: Body surface area
WBC: White blood cell
RFLP: Restriction fragment length polymorphism
ARMS: Tetra-amplification refractory mutation system
PCR: Polymerase chain reaction
TC: Total cholesterol
LDL-C: Low-density lipoprotein cholesterol
HDL-C: High-density lipoprotein cholesterol
TG: Triglycerides
ALT: Alanine aminotransferas
AST: Aspartate aminotransferase
IL6: Interleukin 6
TNFα: Tumor necrosis factorα
hs-CRP: Human high sensitivity C-reactive protein
IFNβ: Interferon β
TAC: Total antioxidant capacity
MDA: Malondialdehyde
8-OHdG: 8-Hydroxy-2′-deoxyguanosine
PAI-1: Plasminogen activator inhibitor-1
MMP9: Matrix metallopeptidase 9
SICAM-1: Soluble intercellular adhesion molecule-1
VCAM-1: Soluble vascular cell adhesion molecule-1
NNFTRI: National Nutrition and Food Technology Research Institute
SBUM: Shahid Beheshti University of Medical Sciences
